# Depression, self-efficacy, and the mediating role of self-esteem: evidence from women entrepreneurs

**DOI:** 10.3389/fpubh.2025.1716599

**Published:** 2026-02-17

**Authors:** Hassan HassanAhmadi, MohammadJawad Hoseinzadeh

**Affiliations:** 1 Faculty of Pharmacy, Shahid Beheshti University of Medical Sciences, Tehran, Iran; 2 Faculty of Administrative and Economics Sciences, Ferdowsi University of Mashhad, Mashhad, Iran

**Keywords:** depression, Herat, PLS-SEM, self-efficacy, self-esteem, women entrepreneurs

## Abstract

This study investigates the relationship between depression and self-efficacy among women entrepreneurs in Herat, with a particular emphasis on the mediating role of self-esteem. The entrepreneurial context, while offering financial independence and personal growth, also exposes women to significant psychological stressors such as financial insecurity, patriarchal norms, and limited social support. A total of 110 entrepreneurial women aged 20–45 participated in this research through convenience sampling. Data were collected using standardized instruments: the Beck Depression Inventory-II (BDI-II), the Coopersmith Self-Esteem Inventory (SEI), and the General Self-Efficacy Scale (GSE). Analysis was performed using Partial Least Squares Structural Equation Modeling (PLS-SEM). Findings revealed that depression negatively predicted both self-esteem and self-efficacy, while self-esteem exerted a significant positive effect on self-efficacy. Mediation testing confirmed that self-esteem partially mediated the relationship between depression and self-efficacy, explaining a substantial proportion of the variance in self-efficacy (*R*^2^ = 0.49). These results highlight the dual psychological challenges faced by women entrepreneurs, where depressive symptoms reduce both perceived competence and self-worth, subsequently lowering efficacy. The study demonstrates that enhancing self-esteem is a key pathway to mitigating the detrimental effects of depression and improving entrepreneurial resilience. Practical implications include the development of interventions such as cognitive-behavioral therapy, self-esteem enhancement workshops, and peer-support initiatives tailored to entrepreneurial contexts. By addressing both depressive symptoms and self-esteem, such strategies may strengthen self-efficacy, support women's psychological well-being, and ultimately promote sustainable entrepreneurial success in developing economies.

## Introduction

Entrepreneurship is recognized as a major driver of productivity and economic development for modern societies (1). Entrepreneurial activity is required to stimulate innovation, create jobs, and produce social life in an innovative economy. However, increased entrepreneurship, like any other occupation, has its advantages and disadvantages. Entrepreneurs are likely to understand that while it benefits, this profession is among the most stressful occupations in the world, and on average, their salaries will not be greater than those of paid employees (1, 2). Yet, most of entrepreneurs report high levels of satisfaction with their life and positive attitudes toward work (1, 3).

The problem statement and research objective: given the increasing psychological and social pressures faced by women entrepreneurs in Herat, the main problem addressed in this study is whether depression reduces their level of self-efficacy and whether self-esteem mediates this relationship. Therefore, the primary objective of this research is to examine the direct effect of depression on self-efficacy and to assess the mediating role of self-esteem in this relationship.

In recent decades, entrepreneurship has attracted increasing attention due to its role in driving social change. Entrepreneurs aim to establish successful businesses through innovation and creativity, and also to make positive contributions toward competition in the labor market (4). This role becomes increasingly important in developing economies, as individuals must work under family demands, gender-based discrimination, and inadequate social support (5). These challenges can cause psychological pressure, making entrepreneurs vulnerable to depression and stress. Empirical studies have shown that entrepreneurs often report high levels of anxiety, loneliness, and stress levels during the early phases of business venture start-ups (6, 7). Given that mental health is a part of human capital, it should be addressed with priority in entrepreneurial ventures, especially for women. Previous research has demonstrated that self-esteem, generalized self-efficacy, neuroticism, and locus of control reflect a shared core psychological construct related to individual adjustment and mental health outcomes (26). Empirical evidence further suggests that self-esteem plays a significant mediating role in the relationship between psychological distress and occupational or academic self-efficacy (27). Additionally, lower levels of self-esteem have been consistently associated with higher depressive symptoms and reduced self-efficacy, particularly among vulnerable populations (28).

The World Health Organization (29) has explained mental health not just as the lack of disease: it is a feeling of well-being that allows individuals to actualize their potential, adapt to life's challenges, be effective in their work, and contribute to society. Mental health, together with the exploration of mental illness, has been emphasized by researchers increasingly, and they have indicated that mental health cannot be dissociated from physical health and behavior (8). Research indicates that women are disproportionately affected by mental illnesses such as depression, anxiety, eating disorders, and post-traumatic stress disorder (30). These are more prevalent in women due to the impact of hormones and life experience (9, 10). Women business owners have brought psychological distress as they need to face patriarchal and competitive business environments. While entrepreneurship may allow financial independence, self-actualization, and self-respect, it is also accompanied by great stressors like financial insecurity and work uncertainty (3, 7).

Of all the psychiatric disorders, depression is among the most pervasive and disabling, affecting interpersonal, occupational, and personal functioning. Among the most notable psychological concepts to influence depression is self-efficacy, meaning individuals' belief in being able to control tasks (11). Empirical findings have established that low self-efficacy increases the vulnerability to depression among women, entrepreneurs, students, and workers (12, 31). Persons who believe they cannot escape adversity experience more helplessness, negative cognitions, and ultimately depression. In contrast, high self-efficacy enhances resilience, emotional control, and motivation and protects against depressive symptoms (13). Self-efficacy-promoting interventions—cognitive-behavioral therapy, coping skills training, and reinforcement counseling—have been successful at alleviating depression symptoms and aiding prevention (14, 15). This issue is particularly relevant to women entrepreneurs, who are compounding professional and societal pressures that raise depression risk unless this robust self-efficacy exists.

Contemporary psychology studies have also underscored self-esteem as a mediating variable in the relationship between self-efficacy and depression. Self-esteem—the level of value that one places upon himself or herself—is the central construct in mental health and influences coping, motivation, and cognitive appraisal (16). Empirical studies confirm that self-efficacy is greater when people have greater self-esteem since a sense of success enhances self-worth (17). At the same time, high self-esteem is also protective against depression: those who are competent and worthy are less likely to feel hopeless and think negatively during adversity (18, 32).

## Method

The target population of this study comprised entrepreneurial women residing in Herat in 2024. One hundred ten participants were recruited using convenience sampling, due to the limited accessibility of women entrepreneurs in the region. While this approach allowed for efficient data collection, it may limit the generalizability of the findings. Inclusion criteria were women actively engaged in entrepreneurial activities, aged between 20 and 45, and willing to provide informed consent. Exclusion criteria included women not currently engaged in entrepreneurship, those outside the specified age range, and incomplete or inconsistent questionnaire responses. Demographic characteristics of the participants were as follows: 38 participants (34.5%) were aged 20–25, 45 participants (40.9%) were 26–30, 19 participants (17.3%) were 31–35, and 8 participants (7.3%) were 36–45 years old. In terms of education, 21 participants (19.1%) had less than a bachelor's degree, 46 (41.8%) held a bachelor's degree, and 43 (39.1%) were undergraduate. Regarding economic status, 20 participants (18.2%) reported a good condition, 47 (42.7%) reported middle, 10 (9.1%) reported excellent, and 33 (30.0%) reported poor economic status. Concerning marital status, 37 participants (33.6%) were single, while 73 (66.4%) were married. Data collection was conducted in person, with participants completing the questionnaires under supervision to ensure accuracy and completeness. Ethical approval was obtained from the relevant local authorities, and informed consent was secured from all participants. Three standardized instruments were used to assess the key psychological constructs: Beck Depression Inventory-II (BDI-II): a 21-item self-report scale measuring the severity of depressive symptoms on a 4-point Likert scale. It demonstrates excellent internal consistency (Cronbach's alpha = 0.91), high test-retest reliability (*r* = 0.93), and strong convergent validity with other depression measures (19). Coopersmith self-esteem inventory: this 25-item yes/no scale evaluates global self-esteem. It has satisfactory internal consistency (Cronbach's alpha 0.68–0.77), good test-retest reliability (0.72–0.85), and established concurrent validity (20). General self-efficacy scale: developed by Jerusalem and Schwarzer, this 10-item, 4-point Likert scale measures individuals' beliefs in their ability to cope with diverse challenges. It demonstrates high internal consistency (Cronbach's alpha 0.76–0.90), good test-retest reliability, and solid convergent validity with related constructs (21). Data were analyzed using SmartPLS 4, which is well-suited for Partial Least Squares Structural Equation Modeling (PLS-SEM).

### Procedure

1. Identification of eligible women entrepreneurs.
2. Obtaining informed consent.
3. Providing a brief explanation of the study's purpose.
4. On-site administration of the questionnaires.
5. Checking the completeness and accuracy of responses.
6. Entering the data into SmartPLS 4.
7. Analyzing the measurement model followed by the structural model.

Justification for PLS-SEM: PLS-SEM was selected due to the relatively small sample size, the exploratory nature of the model, and the partially non-normal distribution of the data.

## Ethical approval

Due to the current political situation in Afghanistan under Taliban control, there are no local authorities authorized to approve research involving women. Therefore, formal ethical approval from an institutional review board was not possible.

Nevertheless, this study was conducted in accordance with the principles of the Declaration of Helsinki, and all participants provided informed consent prior to participation. Participants' confidentiality and safety were strictly maintained throughout the study.

## Results

Pearson correlation analyzes (see Table 1) revealed notable strong positive correlations between General Self-esteem and Family Self-esteem (*r* = 0.93, *p* < 0.01), Social Self-esteem (*r* = 0.92, *p* < 0.01), Professional/Scholastic Self-esteem (*r* = 0.95, *p* < 0.01), and Lie Scale (*r* = 0.92, *p* < 0.01). The Beck Depression Inventory (BDI) showed notable negative correlations with all self-esteem subscales (ranging from *r* = −0.49 to *r* = −0.57, all *p* < 0.01). The Coopersmith Self-Esteem Inventory (SEI) was moderately to strongly positively correlated with the self-esteem subscales (ranging from *r* = 0.54 to *r* = 0.58, *p* < 0.01). Finally, the General Self-Efficacy Scale (EID) demonstrated extremely high positive correlations with all self-esteem dimensions, most notably with General Self-esteem (*r* = 0.99, *p* < 0.01).

**Table 1:** Pearson correlation matrix among study variables (*N* = 110).

**Variable**	**1**	**2**	**3**	**4**	**5**	**6**	**7**	**8**
1. General Self-esteem	1	0.928	0.921	0.950	0.918	−0.558	0.579	0.993
2. Family Self-esteem	0.928	1	0.886	0.912	0.892	−0.485	0.540	0.955
3. Social Self-esteem	0.921	0.886	1	0.881	0.876	−0.567	0.545	0.945
4. Professional/Scholastic Self-esteem	0.950	0.912	0.881	1	0.903	−0.526	0.567	0.966
5. Lie Scale	0.918	0.892	0.876	0.903	1	−0.526	0.562	0.928
6. Beck Depression Inventory	−0.558	−0.485	−0.567	−0.526	−0.526	1	−0.519	−0.556
7. Self-Esteem Inventory	0.579	0.540	0.545	0.567	0.562	−0.519	1	0.580
8. Efficacy Scale	0.993	0.955	0.945	0.966	0.928	−0.556	0.580	1

Descriptive statistics for the study variables are presented in Table 2. The sample size for all variables was 110 with no missing data. The mean scores ranged from 4.19 for the Lie Scale to 30.16 for the Beck Depression Inventory (BDI), with standard deviations between 2.82 (Social Self-esteem) and 17.87 (General Self-Efficacy Scale), indicating substantial variability across measures. Skewness values ranged from −0.17 to 0.156 and kurtosis values from −1.95 to −0.72, all within acceptable limits, suggesting approximate normality. Regarding depression severity, 0.9% of participants were classified as healthy or without depression, 9.1% showed mild depression, 15.5% were in need of consultation, 29.1% experienced moderate depression, 16.4% had relatively severe depression, and 29.1% met the criteria for clinical or severe depression, reflecting a wide distribution of depressive symptoms within the sample.

**Table 2:** Descriptive statistics for study variables (*N* = 110).

**Variable**	***N***	**Mean**	**SD**	**Skewness**	**Kurtosis**
General Self-esteem	110	13.60	9.46	−0.137	−1.893
Family Self-esteem	110	4.20	3.00	−0.131	−1.724
Social Self-esteem	110	4.34	2.82	−0.099	−1.614
Professional/Scholastic SE	110	4.28	3.04	−0.170	−1.751
Lie Scale	110	4.19	3.02	−0.168	−1.672
BDI	110	30.16	11.43	0.122	−1.493
SEI	110	25.51	4.40	0.156	−0.724
EID	110	26.43	17.87	−0.141	−1.950

This study employed Partial Least Squares Structural Equation Modeling (PLS-SEM) to examine the relationship between depression and self-efficacy, with self-esteem serving as a mediating variable. PLS-SEM was selected due to its robustness in handling complex models with relatively small sample sizes and its flexibility regarding data distribution assumptions (22). The hypothesized model included depression as the independent variable, self-esteem as the mediator, and self-efficacy as the dependent variable. The mediating role of self-esteem was assessed to investigate its indirect effect on the relationship between depression and self-efficacy. Measurement model evaluation indicated satisfactory reliability and validity for all constructs. Cronbach's alpha values exceeded 0.8, demonstrating good internal consistency. Composite reliability (rho_a and rho_c) values were above 0.7 for all constructs, confirming construct reliability. Convergent validity was supported for self-esteem (AVE = 0.935) and self-efficacy (AVE = 0.318); depression's AVE was relatively low (0.244), which suggests cautious interpretation but is acceptable for exploratory research (22). Discriminant validity was supported by both the Fornell–Larcker criterion and HTMT values (HTMT < 0.85 for all construct pairs except self-esteem's subdimensions, which is theoretically justifiable due to conceptual overlap). Model fit indices for the estimated model indicated an acceptable fit: SRMR = 0.077, d_ULS = 3.712, d_G = 1.277, Chi-square = 670.365, and NFI = 0.656. While NFI is slightly below the ideal cutoff of 0.90, SRMR and other fit indices suggest that the model provides an adequate representation of the observed data (23).

**Figure 1:**
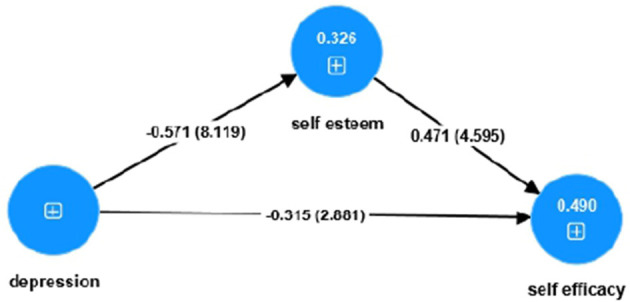
Conceptual model illustrating the direct effect of depression on self-efficacy and the mediating role of self-esteem among women entrepreneurs.

Structural model results revealed that depression had a notable negative direct effect on self-efficacy (β = −0.315, *t* = 2.881, *p* = 0.004) and a notable negative effect on self-esteem (β = −0.571, *t* = 8.119, *p* < 0.001). Self-esteem, in turn, positively influenced self-efficacy (β = 0.471, *t* = 4.595, *p* < 0.001), supporting a partial mediation of self-esteem in the relationship between depression and self-efficacy. The model explained 32.6% of the variance in self-esteem (*R*^2^ = 0.326, adjusted *R*^2^ = 0.319) and 49.0% of the variance in self-efficacy (*R*^2^ = 0.490, adjusted *R*^2^ = 0.481). Stone–Geisser *Q*^2^ values indicated predictive relevance for self-esteem (*Q*^2^ = 0.296) and self-efficacy (*Q*^2^ = 0.141), while depression, as an independent variable, was not expected to be predicted (*Q*^2^ = 0.000) (34, 35). Overall, these findings emphasize the critical mediating role of self-esteem in linking depressive symptoms and self-efficacy. The negative associations between depression and both self-esteem and self-efficacy are consistent with prior literature suggesting that higher depressive symptoms reduce individuals' confidence and self-worth (11, 24). The results underscore the importance of interventions targeting depressive symptoms and promoting self-esteem as effective strategies to enhance self-efficacy, particularly among women entrepreneurs facing multiple professional and social challenges (Figure 1).

Tables 3–6 presents the reliability and validity indices for the constructs used in this study. Cronbach's alpha values ranged from 0.806 to 0.977, indicating good internal consistency across all constructs. Cronbach's alpha measures the extent to which items within a construct are consistent with each other, with values above 0.70 generally considered acceptable (36). Also, composite reliability indices, including rho_a and rho_c, were reported for each construct. Both indices are alternative measures of internal consistency that account for the factor loadings of individual items. Rho_a is a more accurate estimator of reliability based on the PLS algorithm, while rho_c is the traditional composite reliability measure commonly used in SEM. Values above 0.70 indicate that the construct is measured reliably, and all constructs in this study exceeded this threshold (33). The Average Variance Extracted (AVE) was also assessed to examine convergent validity, which reflects the proportion of variance in the items that is explained by the underlying construct. AVE values above 0.50 are generally considered acceptable. In this study, AVE was satisfactory for self-esteem (0.935) and self-efficacy (0.318), although depression showed a relatively low AVE (0.244), suggesting cautious interpretation of its convergent validity. Overall, the reliability and validity analyses indicate that the measurement model is largely robust, but further attention is warranted for the depression construct due to its lower AVE. Convergent validity, assessed via Average Variance Extracted (AVE), was strong for self-esteem, but relatively low for depression and self-efficacy. Low AVE does not automatically imply that a construct is unreliable; rather, it may reflect the complexity and multidimensional nature of the construct, cultural or contextual influences in the sample, or the limitations of the measurement instruments. In this study, the lower AVE for depression and self-efficacy likely reflects the nuanced ways these constructs manifest among entrepreneurial women in Herat, including cultural, linguistic, and situational factors. Importantly, the composite reliability indices were acceptable, supporting the consistency of the constructs despite lower AVE, and making the findings interpretable within an exploratory research context.

**Table 3:** Reliability and validity of constructs.

**Construct**	**Cronbach's alpha**	**Composite reliability (rho_a)**	**Composite reliability (rho_c)**	**Average variance extracted (AVE)**
Depression	0.843	0.85	0.869	0.244
Self-Efficacy	0.43	0.786	0.676	0.318
Self-Esteem	0.977	0.978	0.983	0.935

**Table 4:** Indicator loading and VIF values (Loading + VIF).

**Indicators**	**Depression**	**Self-efficacy**	**Self-esteem**
Depression	1,497	1,000	
Self-efficacy			
Self-esteem	1,497		
BDI1	0.409	−0.231	−0.107
BDI10	0.559	−0.371	−0.318
BDI11	0.396	−0.285	−0.254
BDI12	0.473	−0.32	−0.335
BDI13	0.563	−0.288	−0.359
BDI14	0.502	−0.418	−0.259
BDI15	0.412	−0.199	−0.115
BDI16	0.452	−0.232	−0.29
BDI17	0.554	−0.268	−0.282
BDI18	0.631	−0.45	−0.372
BDI19	0.613	−0.331	−0.402
BDI2	0.963	−0.573	−0.545
BDI20	0.523	−0.325	−0.33
BDI21	0.527	−0.239	−0.359
BDI3	0.478	−0.301	−0.226
BDI4	0.527	−0.326	−0.23
BDI5	0.45	−0.173	−0.248
BDI6	0.453	−0.308	−0.255
BDI7	0.393	−0.175	−0.175
BDI8	0.449	−0.178	−0.245
BDI9	0.466	−0.246	−0.35
EID1	−0.413	0.67	0.413
EID10	−0.536	0.78	0.533
EID11	−0.355	0.708	0.449
EID12	−0.451	0.791	0.463
EID13	−0.472	0.712	0.462
EID14	−0.345	0.599	0.403
EID15	−0.325	0.717	0.513
EID16	−0.348	0.66	0.408
EID17	−0.52	0.834	0.565
EID18	−0.457	0.807	0.519
EID19	−0.538	0.744	0.593
EID2	−0.404	0.694	0.456
EID20	−0.455	0.795	0.471
EID21	−0.508	0.759	0.508
EID22	−0.38	0.809	0.506
EID23	−0.322	0.704	0.439
EID24	−0.306	0.605	0.342
EID25	−0.457	0.822	0.517
EID26	−0.486	0.789	0.48
EID27	−0.465	0.725	0.502
EID28	−0.334	0.66	0.363
EID29	−0.354	0.731	0.464
EID3	−0.357	0.69	0.369
EID30	−0.407	0.702	0.42
EID31	−0.398	0.719	0.406
EID32	−0.419	0.726	0.459
EID33	−0.442	0.718	0.52
EID34	−0.418	0.668	0.543
EID35	−0.457	0.821	0.499
EID36	−0.486	0.753	0.492
EID37	−0.399	0.748	0.478
EID38	−0.468	0.7	0.478
EID39	−0.33	0.563	0.422
EID4	−0.368	0.681	0.487
EID40	−0.456	0.675	0.45
EID41	−0.496	0.719	0.564
EID42	−0.545	0.816	0.542
EID43	−0.506	0.715	0.512
EID44	−0.29	0.556	0.324
EID45	−0.336	0.625	0.38
EID46	−0.376	0.643	0.381
EID47	−0.388	0.686	0.419
EID48	−0.423	0.653	0.512
EID49	−0.296	0.573	0.404
EID5	−0.55	0.674	0.515
EID50	−0.289	0.622	0.368
EID51	−0.43	0.697	0.491
EID52	−0.403	0.766	0.455
EID53	−0.414	0.735	0.499
EID54	−0.358	0.723	0.405
EID55	−0.424	0.741	0.491
EID56	−0.493	0.824	0.576
EID57	−0.331	0.661	0.45
EID58	−0.417	0.75	0.509
EID6	−0.461	0.783	0.515
EID7	−0.455	0.835	0.515
EID8	−0.465	0.648	0.436
EID9	−0.459	0.677	0.498
SEI1	0.494	−0.601	−0.87
SEI10	−0.144	0.209	0.433
SEI2	−0.225	0.401	0.58
SEI3	−0.4	0.261	0.593
SEI4	−0.276	0.381	0.525
SEI5	−0.268	0.298	0.422
SEI6	−0.286	0.25	0.472
SEI7	−0.393	0.395	0.546
SEI8	−0.369	0.392	0.559
SEI9	−0.257	0.381	0.512

**Table 5:** Effect size (*f*^2^) for structural path (*f*^2^ for Depression → SE, Depression → ES → Efficacy).

**Path**	**Depression**	**Self-efficacy**	**Self-esteem**
Depression		0.135	0.497
Self-efficacy			
Self-esteem		0.307	

**Table 6:** Bootstrapped confidence intervals (95%) for structural path (CI lower-upper limits).

**Path**	**Original sample (*O*)**	**Sample mean (*M*)**	**2.50%**	**97.50%**
Depression → Self-efficacy	−0.316	−0.32	−0.517	−0.104
Depression → Self-esteem	−0.576	−0.602	−0.729	−0.486
Self-esteem → Self-efficacy	0.478	0.484	0.288	0.662

Table 7 shows the structural path coefficients, *t*-values, and significance levels for the hypothesized relationships. Results indicate that depression negatively and significantly influences both self-esteem and self-efficacy. Furthermore, self-esteem positively and significantly affects self-efficacy. These findings support the mediating role of self-esteem in the relationship between depression and self-efficacy.

**Table 7:** Structural path analysis results.

**Path**	**β (Original Sample)**	***t*-value**	***p*-value**
Depression → Self-Efficacy	−0.315	2.881	0.004
Depression → Self-Esteem	−0.571	8.119	0.000
Self-Esteem → Self-Efficacy	0.471	4.595	0.000

## Discussion and conclusion

This study investigated the interplay between depression and self-efficacy in entrepreneurial women, specifically examining the mediating role of self-esteem. Updated PLS-SEM analyzes revealed notable negative direct effects of depression on both self-esteem and self-efficacy, as well as a notable indirect effect of depression on self-efficacy through self-esteem. These results confirm self-esteem as a partial mediator in the model. Consistent with prior literature (18, 24), higher levels of depression were associated with lower self-esteem, which in turn decreased self-efficacy. Although some earlier research has suggested that depression might encourage self-reflection or adaptive coping (25), the present findings indicate that, within this sample of entrepreneurial women, depression consistently undermines both self-esteem and self-efficacy. One possible explanation is the cumulative burden of occupational stress, societal expectations, and gender-specific challenges in the entrepreneurial context, which may intensify the negative psychological impact of depressive symptoms. The measurement model demonstrated strong reliability and validity. Cronbach's alpha values ranged from 0.806 to 0.977, indicating high internal consistency. Both composite reliability indices (rho_a and rho_c) exceeded the 0.70 threshold, supporting the reliability of the constructs. Rho_a and rho_c, while similar to Cronbach's alpha, are more tailored to PLS-SEM and take into account the actual factor loadings. Convergent validity was satisfactory for self-esteem (AVE = 0.935) and acceptable for self-efficacy (AVE = 0.318), though the AVE for depression (0.244) was below recommended levels, suggesting caution in interpreting depression-related findings. Discriminant validity, assessed via Fornell–Larcker and HTMT indices, confirmed the constructs' distinctiveness. From a practical standpoint, the results underscore the importance of targeted mental health interventions for entrepreneurial women. Recommended strategies include self-esteem enhancement workshops, skill-building sessions to foster mastery experiences, cognitive-behavioral interventions for managing depressive symptoms, and peer support groups tailored to the entrepreneurial context. Such interventions hold the potential to improve psychological well-being, strengthen self-efficacy, and, by extension, support business performance and sustainability. Nonetheless, several limitations should be acknowledged. The cross-sectional design prohibits causal inference, and the use of convenience sampling, which involves selecting participants who are readily available rather than randomly, may introduce selection bias and limits the generalizability of the findings. Cultural and linguistic factors specific to Herat may have influenced responses, particularly given the self-report method. Additionally, the low AVE for depression points to possible measurement limitations that may affect the robustness of conclusions regarding this construct. Future research should consider longitudinal designs, larger and more diverse samples, culturally adapted measures, and the inclusion of additional mediators or moderators such as social support, coping strategies, or entrepreneurial experience. This study contributes updated empirical evidence regarding the psychological mechanisms affecting entrepreneurial women. It affirms the partial mediating role of self-esteem in the relationship between depression and self-efficacy, highlighting the need for well-designed interventions that address both depressive symptoms and self-esteem. These findings provide practical guidance for fostering mental health and empowering women entrepreneurs, with implications for both individual well-being and professional success.

## Limitations

Several limitations should be acknowledged. First, the low AVE for depression may reflect cultural and linguistic factors affecting interpretation. Second, convenience sampling limits the generalizability of the findings. Third, socio-political constraints in Herat, including restrictions on male researchers interacting with female entrepreneurs in workplaces, restricted access to some participants, potentially influencing sample composition. Additionally, the cross-sectional design prohibits causal inference. Cultural and linguistic factors specific to Herat may have influenced responses, particularly given the self-report method. Future research should consider longitudinal designs, larger and more diverse samples, culturally adapted measures, and the inclusion of additional mediators or moderators such as social support, coping strategies, or entrepreneurial experience. Additionally, the use of self-report instruments may introduce response bias among participants.

## Practical implications

From a practical standpoint, the results underscore the importance of targeted mental health interventions for entrepreneurial women. Recommended strategies include self-esteem enhancement workshops, skill-building sessions to foster mastery experiences, cognitive-behavioral interventions for managing depressive symptoms, and peer support groups tailored to the entrepreneurial context. Such interventions hold the potential to improve psychological well-being, strengthen self-efficacy, and, by extension, support business performance and sustainability. Addressing both depressive symptoms and self-esteem may enhance resilience and empower women entrepreneurs to overcome professional and social challenges.

## Data Availability

The data supporting the findings of this study are available from the corresponding author upon reasonable request.
